# Transactions of the Chicago Gynæcological Society

**Published:** 1879-04

**Authors:** 


### ARTICLE VIII.

5th Meeting, Feb. 28, 1879.

Dr. Nelson read a paper on Subinvolution of the Uterus, published in the present number of the JOURNAL AND EXAMINER.

DR. JONES (speaking 5th) said that his own views had been anticipated by previous speakers.

He could not agree with Dr. Goodell in regard to post-parturient recumbency, and regarded subinvolution as a common condition in females of the lower ranks, due to early rising, and producing in them, as in the more refined, a variety of uterine ailments.

But here the likeness ceased, and differences appeared in the succedanea, due to the exalted nervous susceptibilities found in the latter class. This fact suggested remedial means well known to neuropathologists, i. e., the cultivation of *normal* muscular irritability as a cure for *morbid* irritability of the nervous system.

He had had the best results from the hot water douche, properly applied by Emmet's system, and from the use of ergot.

DR. EARLE gave the history of a case of subinvolution with prolapse and considerable hyperplasia, in which the patient could not, from a variety of causes, present herself for continuous or systematic treatment. The womb measured six inches and was prolapsed so that it made its appearance between the labia. The patient's occupation required constant standing, and the pain was very severe. She was fitted with a Hornby's pessary, and did not report to the doctor for nearly one year. The pessary had been worn all this time with complete relief from all pain, and the uterus now measured 3½ inches. The instrument was repaired and re-adjusted, and the patient told to report again if it caused irritation, or if she did not receive the same relief as before. He had not heard from her since, and supposed her cured.

